# Assessing the protective efficacy of antibodies to the HIV gp41 region by active vaccination

**DOI:** 10.1186/1742-4690-9-S2-P37

**Published:** 2012-09-13

**Authors:** SK Sharma, J Pokorski, MG Finn, J Jacqueline, E Rakasz, D Burton, R Wyatt

**Affiliations:** 1 International AIDS Vaccine Initiative, La Jolla, CA, USA; 2 The Scripps Research Institute, La Jolla, CA, USA; 3 Wisconsin National Primate Research Center, Univ. of Wisconsin-Madison, WI, USA

## Background

The gp41 cluster I is a conserved immunodominant loop connecting the heptad repeat 1 (HR 1) and heptad repeat 2 (HR2) of the HIV-1 the envelope glycoproteins (Env). Following HIV-1 infection or vaccination with gp41-containing Env immunogens, this region elicits relatively high titers of antibodies generally considered to be non-neutralizing in nature. However, in a recent passive immunization study using a cluster 1 antibody, partial protection against SHIV SF162 P4 challenge was observed. In the present study, we sought to determine if vaccine-elicited cluster 1 antibodies might afford some protective capacity by active vaccination, presumably by binding to non-functional spikes on the virus and slowing the viral entry process in vivo.

## Methods

To generate cluster 1-specific antibodies, we added residues flanking the cluster I cysteine-loop region to allow it to assume its preferred structural conformation. The resultant 20 residues peptides were expressed on the genetically modified Q-beta bacteriophage particles and also chemically coupled to KLH. Sera from rabbits immunized with these antigens were analyzed by ELISA for the binding to cluster 1 peptides and by cross-competition with the known cluster I antibodies.

## Results

The cluster I region was found to be immunogenic and, interestingly, a version of the epitope in which alanines were substituted in place of the small cysteine-linked loop was found to be more immunogenic than the wild-type cysteine-cysteine motif. The sera from rabbits inoculated with either carrier cross-competed with the known cluster I antibodies such as F240. Though the sera did not neutralize JR-FL viruses, they serum antibodies were able to capture many different viruses in vitro.

## Conclusion

We conclude that we have specifically elicited antibodies directed to the cluster 1 region of gp41 possessing properties similar to the known monoclonal antibodies. Active immunization of non-human primates by both the intranasal and intramuscular routes, followed by SHIV.

